# The presence and relative frequency detection of the levamisole-resistance-associated S168T substitution in *hco-acr-8* in *Haemonchus contortus*

**DOI:** 10.1016/j.ijpddr.2023.02.002

**Published:** 2023-02-08

**Authors:** Paulius Baltrušis, Peter Halvarsson, Claude L. Charvet, Johan Höglund

**Affiliations:** a Department of Biomedical Sciences and Veterinary Public Health, Section for Parasitology, Swedish University of Agricultural Sciences, Uppsala, Sweden; b INRAE, Université de Tours, ISP, 37380, Nouzilly, France

**Keywords:** S168T, Levamisole resistance, Anthelmintic resistance, Droplet digital PCR, Allele-specific PCR, acr8

## Abstract

Parasitic sheep nematodes, among which *Haemonchus contortus* is often considered to be the most clinically important, exact a significant toll on the animals, not least because of their capacity to evolve drug resistance. Despite decades of research, our understanding of the mechanism of resistance to compounds such as levamisole is fairly limited, which therefore constrains our ability to develop sensitive and efficient molecular diagnostic tools for rapid and accurate resistance detection in field settings. Herein, we investigated the presence and frequency of the newly reported, levamisole-resistance-associated, mutation, yielding a S168T substitution in exon 4 of *hco-acr-8*, in six different phenotypically described isolates (three susceptible and three resistant), three Swedish field isolates and eight larvae culture samples, the latter two of which originated on farms where levamisole showed complete parasite elimination. For this purpose, we created both an allele-specific and droplet digital PCR approaches and found the mutated allele to be present only in the Kokstad isolate, whereas the other five as well as both the Swedish isolates and larvae cultures displayed only the non-mutated, serine-encoding, allele. While the finding of only the non-mutated allele in the phenotypically susceptible and Swedish isolate and larvae culture samples seemed sensible, we speculate that for the other two phenotypically resistant isolates, different (perhaps secondary) variants are responsible for conferring the resistance to levamisole phenotype, given the polygenic nature of levamisole resistance. All in all, despite the limited number of samples tested here, the mutation causing the S168T substitution in *hco-acr-8* represents a plausible levamisole resistance-associated variant in, at least, some isolates of *H. contortus*.

## Highlights

• S168T mutation studied in mostly lev-susceptible *H. contortus* isolates and cultures.
• Allele-specific and droplet digital PCR yielded identical results for the isolates.
• Mutated allele found only in the lev-resistant Kokstad isolate worms.
• Unmutated alleles found in lev-susceptible Swedish isolates and larvae cultures.
• S168T mutation could be a good marker for lev-resistance in some populations.

## Graphical abstract

**Figure:**
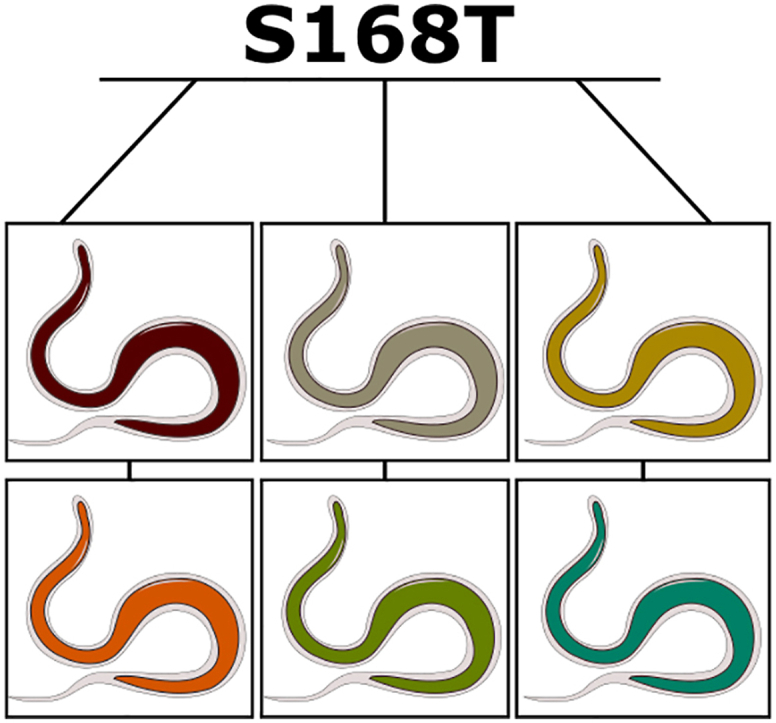

## 1 Introduction

Parasitic nematodes contribute to the deterioration of animal health, which in turn affects their welfare and the productivity of the livestock farming industry (Charlier et al., 2020). In the small ruminant sector, one of the most pathogenic, and thus important, parasites is *Haemonchus contortus*. Over time this species has demonstrated the capacity to develop resistance to all currently available drug classes, in some cases extremely rapidly (Höglund et al., 2020). Although the development of anthelmintic resistance in parasites is not a recent issue, apart from benzimidazoles, little conclusive evidence has been presented to delineate the major, causal genetic factors giving rise to populations of parasites resistant to other drugs, such as levamisole. What is more, unlike benzimidazoles and ivermectin and due to its seldom use, levamisole is still a very efficacious drug in some countries (e.g., Sweden) (Höglund et al., 2022). Therefore, despite the current limitations in carrying out efficient and sensitive, molecular-based screenings, it is of utmost importance that its potency is preserved through careful but rigorous monitoring for levamisole resistance in parasite populations.

Up until recently, the prevailing hypothesis regarding the major mechanism responsible for levamisole resistance in *H. contortus* was based on the observation that some phenotypically resistant isolates appeared to express a truncated transcript of *hco-acr-8* (hco-acr-8b) as a result of the (63bp) deletion in the intron 2 (Fauvin et al., 2010; Barrère et al., 2014). However, growing interest in *hco-acr-8* and its role in resistance has resulted in findings inconsistent with this hypothesis (Chagas et al., 2016; Baltrušis et al., 2021; Doyle et al., 2022). The most recent data suggests that a non-synonymous mutation (A**G**C→A**C**C; protein coding strand) in the exon 4 of this gene, resulting in the S168T substitution, could, instead, be the major determinant of levamisole resistance and serve as a potential molecular marker of its identification in parasite populations (Doyle et al., 2022; Antonopoulos et al., 2022).

In this study, we aimed to analyze the samples at our disposal from the previous study (Baltrušis et al., 2021) with a newly developed allele-specific PCR (AS-PCR) and droplet digital (dd) PCR approaches to investigate the presence or absence of the mutation A**G**C→A**C**C at codon position 168 (hereafter referred to as mutation S168T) in *hco-acr-8* in six phenotypically characterized and three Swedish, field *H. contortus* isolates. In addition, the ddPCR assay was also employed to perform relative frequency analyses of the mutation S168T using genomic DNA from locally recovered larvae cultures (where levamisole was shown to be 100% efficacious). In this way, we hoped to not only contribute to the development of a quantitative test to determine the frequency of the S168T allele, possibly associated with levamisole resistance in *H. contortus*, but to also attempt to elucidate the connection between this allele and the phenotypic (levamisole resistance) status of individual and larvae populations of *H. contortus*.

## 2 Materials and methods

### 2.1 Sample origins

The samples used in this study have been described in our previous work (Baltrušis et al., 2021). Briefly, in order to establish the presence or absence and frequencies of the mutation S168T in *hco-acr-8* (HCON_00151270), genomic DNA (for extraction procedures please refer to Baltrušis et al., 2021), belonging to individual, adult *H. contortus* and mostly *H. contortus* larvae populations (see Supplementary Table 1) was used. The larvae population samples were previously collected as fecal samples (∼2g of feces per animal; 10–15 animals per larvae culture sample) on farms where levamisole was shown to be fully efficacious through both FECRT and molecular testing. It is however important to note that, due to already low egg counts in the pre-treatment sample taken on farm F8, the post-treatment sample was not collected. Thus, as regards to farm F8 - the efficacy of the treatment was implied rather than properly tested.

### 2.2 Allele-specific PCR

AS-PCR was developed to investigate the susceptible and resistant isolates by manually designing two forward primers, 5′TCTAAGAGGAATCCATTGTCG**C**3′ and 5′TCTAAGAGGAATCCATTGTCG**G**3′, and a universal reverse primer - 5′CCGATGGTGAGCCTCATATTACA3’ (yielding amplicon sizes of 184bp; the principle has been described by Chen and Schedl, 2021) using the exon 4 sequence (of *hco-acr-8*) information, available by examining the most recently updated *H. contortus* genome assembly (MHCO3ISE_4.0) (Doyle et al., 2020) on the WormBase ParaSite domain (parasite.wormbase.org/index.html). Amplification was carried out using the AmpliTaq Gold™ DNA Polymerase kit (ThermoFisher Scientific) and a standard 40-cycle PCR protocol: a single cycle of 95 °C for 5 min, 40 cycles of 95 °C for 45 s, 30 s of 56 °C, and 1 min of 72 °C, followed by a single extension cycle of 72 °C for 10 min. The final concentrations of PCR-reagents were: 1X PCR buffer, 0.2 mM of dNTP mix, 1.5 mM of MgCl_2_, 1 μM of each primer and 1.25 U of polymerase. A fixed volume of 3 μl of template was used per amplification reaction, while the total, per reaction gDNA input never exceeded the <1 μg recommendation, indicated by the manufacturers of the polymerase kit. The amplification products were visualized using GelRed® dye on a 2% agarose gel.

### 2.3 Droplet digital PCR

Using the output from the AS-PCR as a reference, a droplet digital PCR approach was created for the simultaneous detection and quantification of both the A**G**C (hereafter S; susceptibility-associated) and A**C**C (hereafter R; resistance-associated) alleles in our samples. The forward and reverse primers (5′GGTAACTGCCGCACATCTAA3′, 5′CTACAAATCATTCTGTCCAATCAATA3′) and probes (5′FAM-TGGAGGATGGA**G** CTACAATGGATTCCT-IowaBlackFQ3′ and 5′HEX-TGGAGGATGGA**C**CTACAATGGATTCCT-IowaBlackFQ3′), generating and detecting 117bp long amplicons, were created *in silico* and tested on the same six isolates, in addition to the Swedish (adult worm) field isolates and larvae population samples. The protocol, together with the annealing temperature, for ddPCR, was identical to the one described in our previous study (Baltrušis et al., 2021). We set the manual thresholds to distinguish the droplet clusters at 9000 fluorescence amplitude units (FAU) for the FAM-labelled (S allele detecting) probe and 4000 FAU for the HEX-labelled (R allele detecting) probe. Since negative template control samples (N) were included in every run and consistently yielded no false positives, >1 positive droplet above the aforementioned threshold(s) was considered to be indicative of the presence of the respective allele(s) in the sample.

### 2.4 Data visualization

Amplicon copy and relative allele frequency data was visualized using the packages *ggplot2* (3.3.6; github.com/tidyverse/ggplot2) and *patchwork* (1.1.2; cloud.r-project.org/web/packages/patchwork/index.html) in R (4.2.1).

## 3 Results and discussion

We utilized both AS-PCR and ddPCR to genotype the *H. contortus* worms belonging to the six phenotypically characterized isolates, in terms of the mutation S168T in *hco-acr-8*.

**Fig. 1:**
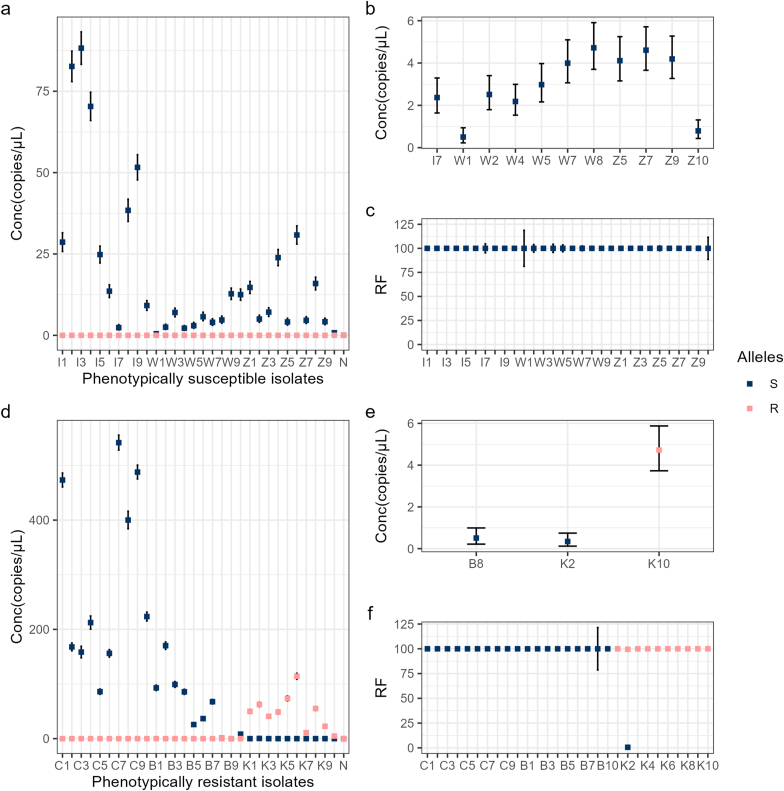
Concentration (amplicon copy numbers per microliter) and relative frequencies (RF) of the S (AGC) and R (ACC) alleles in (codon position 168; exon 4) *hco-acr-8*. (**a**) The concentration of S (susceptibility-associated; AGC) and R (resistance-associated; ACC) alleles for isolates *ISE*, *Weybridge,* and *Zaire* (n = 10 each). (**b**) Samples from (**a**) in which the concentration of alleles was <5 copies per microliter. (**c**) Relative frequency (RF) of the S allele in the samples from (**a**). (**d**) The concentration of S and R alleles for isolates *Cedara*, *Borgsteede* (no signal was found for B9)*,* and *Kokstad* (n = 10 each). (**e**) Samples from (**d**) in which the concentration of alleles was <5 copies per microliter. Relative frequency (RF) of the S and R alleles in the samples from (**d**). (N=Negative template control).

Only S alleles were identified for all six isolates, except for the individuals belonging to the Kokstad isolate group, among which nine were homozygous for the R allele and one - heterozygous (K2; Supplementary Fig. 1). Similarly, using the ddPCR approach, we identified only the S allele (0.50 copies/μl - 88.24 copies/μl in the phenotypically susceptible isolates and 0.34 copies/μl - 487.95 copies/μl in the phenotypically resistant isolates; the signal for sample B9 was not detected), except for samples belonging to the Kokstad isolate category (Fig. 1). Within the latter isolate, the R allele was found to be ranging between 0.34 copies/μl and 541.83 copies/μl. In agreement with AS-PCR, the K2 sample was confirmed to be heterozygous, despite both the copy number (0.34 copies/μl) and relative frequency (0.54%) of the S allele being much lower than that of the R allele. These findings are further supported by Doyle et al. (2022), who have recently confirmed that in their previous study on the global diversity of *H. contortus* (Sallé et al., 2019), the Kokstad isolate, unlike others, had also demonstrated fixed frequencies of the mutation S168T.

Although unexpected, the fact that both *Cedara* and *Borgsteede* isolates, which are phenotypically resistant to levamisole, possessed only the S allele could be the consequence of the limited number of samples tested for each of the isolates (n = 10 each). Therefore, given that levamisole resistance is a quantitative trait (Sangster et al., 1998) and that individuals within a resistant population are distributed continuously in terms of their individual levels of resistance (Clec'h et al., 2021), it is plausible that the small subsets of these two populations were not strictly of resistant phenotype but, rather, survived selection due to other unrelated reasons. Alternatively, and perhaps more plausibly, other, secondary resistance-conferring variants could have contributed to resistance development in these isolates, much like multiple allele variants determine the resistance phenotype in different benzimidazole-resistant *H. contortus* isolates (Baltrušis et al., 2020). For example, in the *Borgsteede* isolate, resistance has previously been linked to changes in a different gene – *hco-unc-63* (Neveu et al., 2010; Boulin et al., 2011)*.* Furthermore, this hypothesis is also reinforced by taking note of the differences in, for example, the EC50 values between the *Borgsteede* and Kokstad isolates to levamisole, which are approximately 621 μg/ml (∼3000 μM) and 14.01 μM, respectively (Hoekstra et al., 1997; Charvet et al., 2018). Thus, this observation would indicate that the *Borgsteede* isolate is on average 214-times more resistant to levamisole than the Kokstad isolate, despite the individuals belonging to the former only possessing the S allele in this study. However, it is important to note that these values should be interpreted with caution as they were reported more than 20 years apart from one another, in addition to being determined using different *in vitro* approaches.

**Fig. 2:**
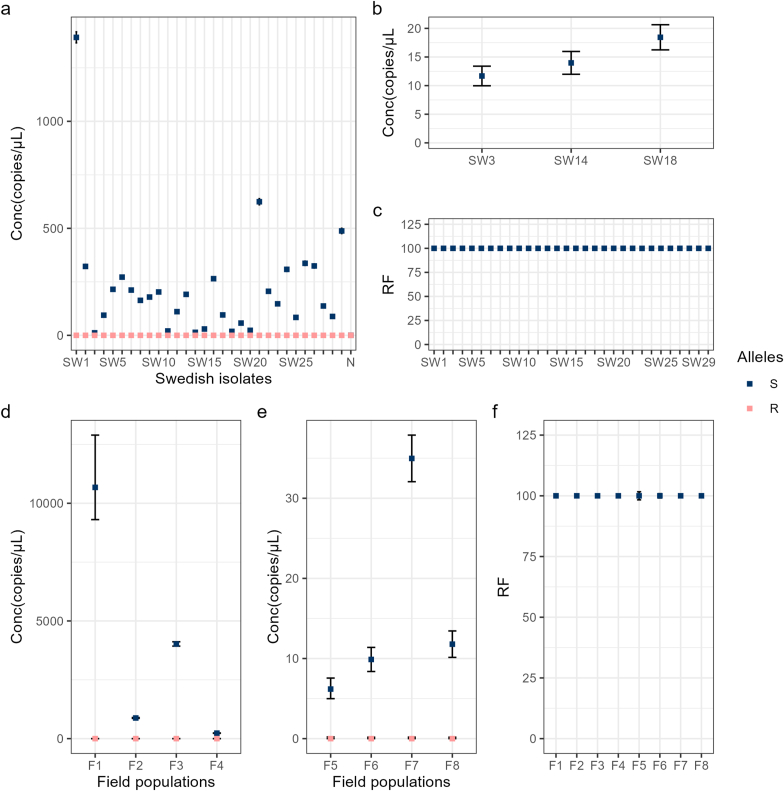
Concentration (amplicon copy numbers per microliter) and relative frequencies (RF) of S (AGC) and R (ACC) alleles in (codon position 168; exon 4) *hco-acr-8*. (**a**) The concentration of S (susceptibility-associated; AGC) and R (resistance-associated; ACC) alleles for isolates *A2018* (SW1-SW10), *A2020* (SW11-20)*,* and *B* (SW21-SW30). (**b**) Samples from (**a**) in which the concentration of alleles was <20 copies per microliter. (**c**) Relative frequency (RF) of the S allele in the samples from (**a**). (**d** and **e**) The concentration of S and R alleles in larvae populations, initially recovered as fecal samples, on 8 farms. (**f**) Relative frequency (RF) of the S and R alleles in the samples from (**d** and **e**). (N=Negative template control).

In addition to the six, phenotypically described isolates, we investigated the frequencies of S and R alleles in three Swedish field isolates, recovered on farms where levamisole was shown to be efficacious in the past (Fig. 2). Unsurprisingly, all 30 individuals possessed only the S allele.

Having established the genotypes of the single worm isolates, we attempted to determine the frequencies of the R allele in larvae populations, collected on sheep farms where levamisole was also shown to be fully efficacious (Fig. 2). In contrast to our previously published data in Baltrušis et al. (2021), only the S allele was found in these populations, which would in turn suggest drug susceptibility if the presence of the secondary variants was to be ignored. This observation is further reinforced by both the FECRT, and previous molecular testing done on most of these samples. Thus, as regards *hco-acr-8* and its involvement in levamisole resistance in the parasite *H. contortus*, in our estimation, the data presented for S168T is so far more consistent with this mutation being a likely causal or closely-linked variant, associated with resistance, in comparison to the deletion in intron 2, which was readily found in both phenotypically susceptible and resistant isolates/populations (Chagas et al., 2016; Baltrušis et al., 2021; Doyle et al., 2022; Antonopoulos et al., 2022). However, it should also be noted that S168T alone is unlikely to explain all cases of phenotypic resistance.

Finally, it is important to highlight that due to the polymorphic nature of *H. contortus* genome, amplification assays are likely to encounter issues related to amplification efficiency, especially in the more mutation-prone regions (as discussed in Baltrušis et al., 2021), which can bias the efforts to identify the precise frequencies of each allele in question. Therefore, although we utilized the publicly available reference genome to develop primers and probes for both of our assays, population wide sequencing is a preferable approach to identify and take into account the total genetic variation within the region of interest when designing the primers and to a degree, probe sequences.

In conclusion, we have successfully applied ddPCR to detect and estimate the relative frequency of the most recently reported S168T (A**G**C→A**C**C) mutation, linked to levamisole resistance, to study multiple (phenotypically) different *H. contortus* isolates and field larvae populations recovered from Swedish sheep farms that underwent treatment with levamisole, in addition to confirming our results with those obtained from an independent AS-PCR.

## Declaration of competing interest

The authors of this manuscript certify that they have NO affiliations with or involvement in any organization or entity with any financial interest, or non-financial interest in the subject matter discussed in this manuscript.

## References

[bib1] Antonopoulos A., Doyle S.R., Bartley D.J., Morrison A.A., Kaplan R., Howell S., Neveu C., Busin V., Devaney E., Laing R. (2022). Allele specific PCR for a major marker of levamisole resistance in Haemonchus contortus. Int. J. Parasitol. Drugs Drug Resist..

[bib2] Baltrušis P., Charvet C.L., Halvarsson P., Mikko S., Höglund J. (2021). Using droplet digital PCR for the detection of hco-acr-8b levamisole resistance marker in H. contortus. Int. J. Parasitol. Drugs Drug Resist..

[bib3] Baltrušis P., Komáromyová M., Várady M., von Samson-Himmelstjerna G., Höglund J. (2020). Assessment of the F200Y mutation frequency in the β tubulin gene of Haemonchus contortus following the exposure to a discriminating concentration of thiabendazole in the egg hatch test. Exp. Parasitol..

[bib4] Barrère V., Beech R.N., Charvet C.L., Prichard R.K. (2014). Novel assay for the detection and monitoring of levamisole resistance in Haemonchus contortus. Int. J. Parasitol..

[bib5] Boulin T., Fauvin A., Charvet C.L., Cortet J., Cabaret J., Bessereau J.L., Neveu C. (2011). Functional reconstitution of Haemonchus contortus acetylcholine receptors in Xenopus oocytes provides mechanistic insights into levamisole resistance. Br. J. Pharmacol..

[bib6] Chagas A.C. de S., Domingues L.F., Gaínza Y.A., Barioni-Júnior W., Esteves S.N., Niciura S.C.M. (2016). Target selected treatment with levamisole to control the development of anthelmintic resistance in a sheep flock. Parasitol. Res..

[bib7] Charlier J., Rinaldi L., Musella V., Ploeger H.W., Chartier C., Vineer H.R., Hinney B., von Samson-Himmelstjerna G., Băcescu B., Mickiewicz M., Mateus T.L., Martinez-Valladares M., Quealy S., Azaizeh H., Sekovska B., Akkari H., Petkevicius S., Hektoen L., Höglund J., Morgan E.R., Bartley D.J., Claerebout E. (2020). Initial assessment of the economic burden of major parasitic helminth infections to the ruminant livestock industry in Europe. Prev. Vet. Med..

[bib8] Charvet C.L., Guégnard F., Courtot E., Cortet J., Neveu C. (2018). Nicotine-sensitive acetylcholine receptors are relevant pharmacological targets for the control of multidrug resistant parasitic nematodes. Int. J. Parasitol. Drugs Drug Resist..

[bib9] Chen J., Schedl T. (2021). A simple one-step PCR assay for SNP detection. MicroPublication Biol.

[bib10] Clec’h W.L., Chevalier F.D., Mattos A.C.A., Strickland A., Diaz R., McDew-White M., Rohr C.M., Kinung’hi S., Allan F., Webster B.L., Webster J.P., Emery A.M., Rollinson D., Djirmay A.G., Al Mashikhi K.M., Al Yafae S., Idris M.A., Moné H., Mouahid G., LoVerde P., Marchant J.S., Anderson T.J.C. (2021). Genetic analysis of praziquantel response in schistosome parasites implicates a transient receptor potential channel. Sci. Transl. Med..

[bib11] Doyle S.R., Laing R., Bartley D., Morrison A., Holroyd N., Maitland K., Antonopoulos A., Chaudhry U., Flis I., Howell S., McIntyre J., Gilleard J.S., Tait A., Mable B., Kaplan R., Sargison N., Britton C., Berriman M., Devaney E., Cotton J.A. (2022). Genomic landscape of drug response reveals mediators of anthelmintic resistance. Cell Rep..

[bib12] Doyle S.R., Tracey A., Laing R., Holroyd N., Bartley D., Bazant W., Beasley H., Beech R., Britton C., Brooks K., Chaudhry U., Maitland K., Martinelli A., Noonan J.D., Paulini M., Quail M.A., Redman E., Rodgers F.H., Sallé G., Shabbir M.Z., Sankaranarayanan G., Wit J., Howe K.L., Sargison N., Devaney E., Berriman M., Gilleard J.S., Cotton J.A. (2020). Genomic and transcriptomic variation defines the chromosome-scale assembly of Haemonchus contortus, a model gastrointestinal worm. Commun. Biol..

[bib13] Fauvin A., Charvet C., Issouf M., Cortet J., Cabaret J., Neveu C. (2010). cDNA-AFLP analysis in levamisole-resistant Haemonchus contortus reveals alternative splicing in a nicotinic acetylcholine receptor subunit. Mol. Biochem. Parasitol..

[bib14] Hoekstra R., Borgsteede F.H.M., Boersema J.H., Roos M.H. (1997). Selection for high levamisole resistance in Haemonchus contortus monitored with an egg-hatch assay. Int. J. Parasitol..

[bib15] Höglund J., Baltrušis P., Enweji N., Gustafsson K. (2022). Signs of multiple anthelmintic resistance in sheep gastrointestinal nematodes in Sweden. Vet. Parasitol. Reg. Stud. Rep..

[bib16] Höglund J., Enweji N., Gustafsson K. (2020). First case of monepantel resistant nematodes of sheep in Sweden. Vet. Parasitol. Reg. Stud. Rep..

[bib17] Neveu C., Charvet C.L., Fauvin A., Cortet J., Beech R.N., Cabaret J. (2010). Genetic diversity of levamisole receptor subunits in parasitic nematode species and abbreviated transcripts associated with resistance. Pharmacogenetics Genom..

[bib18] Sallé G., Doyle S.R., Cortet J., Cabaret J., Berriman M., Holroyd N., Cotton J.A. (2019). The global diversity of Haemonchus contortus is shaped by human intervention and climate. Nat. Commun..

[bib19] Sangster N.C., Redwin J.M., Bjorn H. (1998). Inheritance of levamisole and benzimidazole resistance in an isolate of Haemonchus contortus. Int. J. Parasitol..

