# Supplementary material for: The presence and relative frequency detection of the levamisole-resistance-associated S168T substitution in hco-acr-8 in Haemonchus contortus
Source: Int J Parasitol Drugs Drug Resist. 2023 Feb 8;21:91–5. doi: 10.1016/j.ijpddr.2023.02.002 (PMC9945773; doi:10.1016/j.ijpddr.2023.02.002)
Supplement: Multimedia component 1 [file mmc1.docx]

| \| **Sample batch number** \| **Isolate name** \| **Parasitic life stage** \| **Resistance status** \| **Reference** \| **Number tested** \| **AS-PCR** \| **ddPCR** \| \| --- \| --- \| --- \| --- \| --- \| --- \| --- \| --- \| \| 1 \| *ISE* \| adult \| Susceptible \| Barrère et al., 2014 \| 10 \| Yes \| Yes \| \| 2 \| *Weybridge* \| adult \| Susceptible \| Neveu et al., 2010 \| 10 \| Yes \| Yes \| \| 3 \| *Zaire* \| adult \| Susceptible \| Fauvin et al., 2010 \| 10 \| Yes \| Yes \| \| 4 \| *Cedara* \| adult \| Resistant \| Barrère et al., 2014 \| 10 \| Yes \| Yes \| \| 5 \| *Borgsteede(RHS6)* \| adult \| Resistant \| Hoekstra et al., 1997 \| 10 \| Yes \| Yes \| \| 6 \| *Kokstad* \| adult \| Resistant \| Fauvin et al., 2010 \| 10 \| Yes \| Yes \| \| 7 \| *A2018* \| adult \| Susceptible \| Baltrušis et al., 2021 \| 10 \| No \| Yes \| \| 8 \| *A2020* \| adult \| Susceptible \| Baltrušis et al., 2021 \| 10 \| No \| Yes \| \| 9 \| *B* \| adult \| Susceptible \| Baltrušis et al., 2021 \| 10 \| No \| Yes \| \| 10 \| *F1 or Farm A (Baltrušis et al., 2021)* \| L3 \| Susceptible \| Baltrušis et al., 2021 \| Pooled pre-treatment population \| No \| Yes \| \| 11 \| *F2 or Farm B (Baltrušis et al., 2021)* \| L3 \| Susceptible \| Baltrušis et al., 2021 \| Pooled pre-treatment population \| No \| Yes \| \| 12 \| *F3 or Farm C (Baltrušis et al., 2021)* \| L3 \| Susceptible \| Baltrušis et al., 2021 \| Pooled pre-treatment population \| No \| Yes \| \| 13 \| *F4 or Farm D (Baltrušis et al., 2021)* \| L3 \| Susceptible \| Baltrušis et al., 2021 \| Pooled pre-treatment population \| No \| Yes \| \| 14 \| *F5 or Farm E (Baltrušis et al., 2021)* \| L3 \| Susceptible \| Baltrušis et al., 2021 \| Pooled pre-treatment population \| No \| Yes \| \| 15 \| *F6 or Farm F (Baltrušis et al., 2021)* \| L3 \| Susceptible \| Baltrušis et al., 2021 \| Pooled pre-treatment population \| No \| Yes \| \| 16 \| *F7* \| L3 \| Susceptible \| - \| Pooled pre-treatment population \| No \| Yes \| \| 17 \| *F8 or Farm G (**Baltrušis et al., 2021)* \| L3 \| Not known* \| Baltrušis et al., 2021 \| Pooled pre-treatment population \| No \| Yes \| |
| --- | --- | --- | --- | --- | --- | --- | --- | --- | --- | --- | --- | --- | --- | --- | --- | --- | --- | --- | --- | --- | --- | --- | --- | --- | --- | --- | --- | --- | --- | --- | --- | --- | --- | --- | --- | --- | --- | --- | --- | --- | --- | --- | --- | --- | --- | --- | --- | --- | --- | --- | --- | --- | --- | --- | --- | --- | --- | --- | --- | --- | --- | --- | --- | --- | --- | --- | --- | --- | --- | --- | --- | --- | --- | --- | --- | --- | --- | --- | --- | --- | --- | --- | --- | --- | --- | --- | --- | --- | --- | --- | --- | --- | --- | --- | --- | --- | --- | --- | --- | --- | --- | --- | --- | --- | --- | --- | --- | --- | --- | --- | --- | --- | --- | --- | --- | --- | --- | --- | --- | --- | --- | --- | --- | --- | --- | --- | --- | --- | --- | --- | --- | --- | --- | --- | --- | --- | --- | --- | --- | --- | --- | --- | --- | --- |
