# Supplementary figures and images for: The presence and relative frequency detection of the levamisole-resistance-associated S168T substitution in hco-acr-8 in Haemonchus contortus
Source: Int J Parasitol Drugs Drug Resist. 2023 Feb 8;21:91–5. doi: 10.1016/j.ijpddr.2023.02.002 (PMC9945773; doi:10.1016/j.ijpddr.2023.02.002)

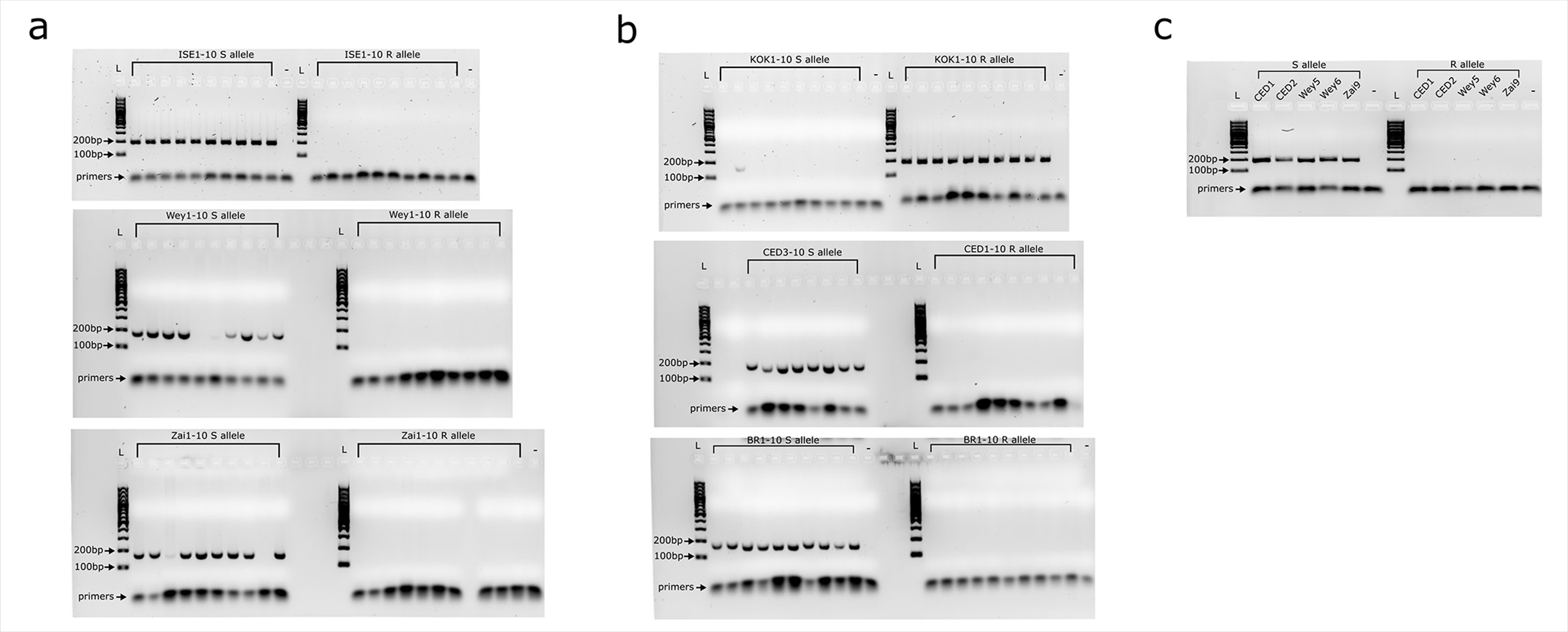

Supplement: Fig. S1 [file mmcfigs1.jpg]
